# The Question of Dental Infection in the Production of Nervous and Mental Diseases

**Published:** 1920-04

**Authors:** Charles K. Mills

**Affiliations:** Philadelphia, Pa.


### THE QUESTION OF DENTAL INFECTION IN THE PRODUCTION OF NERVOUS AND MENTAL DISEASES

BY CHARLES K. MILLS, M.D., PHILADELPHIA, PA.

Fortunately, the mind of the profession seems to be tending toward a very healthy discussion of the probable fallacies of dental infection in its relation to various important diseases, and of many other correlated and zealously exploited views. Shortly after the death of Colonel Roosevelt a newspaper article appeared which illustrated one of the most dangerous forms of medical propaganda. It was stated that one of Colonel Roosevelt's teeth was the indirect cause of his death, and that thousands of persons die or are incapacitated yearly from maladies arising from infected teeth. The cases of a rescued backward boy whose brain was clouded to the point at which he could not master the rudiments of knowledge, and a youth assailed with a lethargy at the age of 15, who afterward became one of the most brilliant lawyers of the Middle West, were included in a series of cases supposedly illustrative of the marvelous results of the removal of infected teeth. *I have but little doubt that literally bushels of teeth of excellent quality have been sacrificed as the result of this atrocious article about Colonel Roosevelt.* Viewing roentgenograms, I have been skeptical as to what are designated as abscesses at the roots of teeth. It has always seemed to me that these pictures represented absorptive lacunae or artefacts of some other description, rather than true abscesses. The roentgen ray enables us to discriminate between areas of differing density. In a strict sense an abscess can not be demonstrated by a roentgenologic investigation. *A score or more of cases have come to my knowledge in which important mental and nervous diseases have been attributed to dental infection,*

*and in which the teeth had been removed with results in some instances so harmful as to make me feel that the procedure was almost a criminal one.* Dr. Cotton, one of the leading psychiatrists of the country, attributes many and diverse forms of insanity to dental infection. The late Dr. August Hoch, in a critical review of one of Dr. Cotton's articles, clearly points out the logical defects in the presentation. Under the influence of the propaganda of focal infection, to use the expression of Dr. Peterson, the colons of epileptics bid fair to be reduced to semicolons by operation. The appendix will soon no longer be a vestigial illustration, and the tonsil, protrusive and submerged, is sharing the fate of the ovary in our early experience. A nose and throat specialist not long ago gravely informed me that he thought of preparing a statute to be presented to the state legislature making compulsory the extirpation of the tonsils of children after reaching a certain age, on the theory that prevention is better than cure. On the same principle may not our exodontist friends be called in for their exterminating activities, and thus free the rising generation of teeth which in the course of time may have their roots infected and abscessed? *I protest against the too free use of the therapeutics of organic mutilation. If the craze for violent removal goes on, it will come to pass that we shall have a gutless, glandless, toothless, and perhaps, thanks to psychology and surgery, a witless race.*

### DENTAL THERAPEUTICS BASED ON CLINICAL AND ROENTGEN-RAY INVESTIGATIONS

*William Middleton Fine*

*As a dentist I am forced to believe that too many teeth are extracted in the expectation that their removal will cure systemic disturbances.* Because it is possible to demonstrate the same micro-organisms in pulpless teeth and in arthritic joints, it should not be stated that the teeth are invariably the primary cause of the infection.

The extraction of badly decayed teeth with root abscesses, or their restoration to health and usefulness, removes one of the contributing factors in the cause and development of many diseases. *I do not share the belief that all non-vital teeth should be removed.* Our bodily protective processes prevent well-treated teeth, without living pulps, being the direct cause of disease. Talbot would lead us to believe that all the shadows in roentgenograms of dead teeth are not abscesses, and that these teeth should not be extracted ruthlessly. I agree with those who advise the extraction of dead and diseased teeth after a thorough attempt to effect a cure has failed or is doubtful. It is more than likely that 75 per cent. of the shadows in the roentgenograms at the apical ends of teeth have been caused by irritation from mechanical treatment and the use of drugs in dental operations, and hence these teeth are removed unjustifiably. I have seen teeth that roentgen-ray examination indicated were abscessed—as some men would say—remain fifteen years or longer without any symptom of disease, local or general. *I received recently three cards from men announcing that they were giving up general practice to specialize in extraction of teeth. We do not need three new extractors every week.*

### PRESENT STATUS OF ORAL SEPSIS IN RELATION TO SYSTEMIC DISEASE

*James M. Anders*

The importance of the relation of tooth root infection to systemic diseases can not be overemphasized from the point of view of study and investigation. Our knowledge of the flora of the mouth, as related to special affections, is still imperfect. A closer co-operation between dentist and physician along bacteriologic lines is urgently needed to deal intelligently with affections of the teeth and the alveolar processes, and to correct the haphazard sacrifice of the masticating apparatus. I recall a case, one of epilepsy, in a young woman, aged 19, in which, on the advice of a physician, all the teeth, although in good condition, were extracted, without, of course, any effect on the epilepsy. It behooves physicians and dentists to oppose the appalling present rate of removal of teeth. It has been shown that after the fortieth year of life, disseminated infections leading to constitutional disturbances are more frequent and of more serious character than in younger subjects. That many morbid medical conditions may be of oral origin is shown in their cure following the removal of the foci of oral infection. Among many such medical conditions are arthritis, endocarditis, pericarditis, myocarditis, gastritis, myocardial degeneration, chronic nephritis and exophthalmic goiter. Gingival and dental diseases, however, are rarely the sole cause of the morbid states for which gums and teeth may be held responsible. Before teeth are condemned, it is a matter of vital importance to determine that other than oral infectious foci are not present. Treatment based on roentgen-ray studies alone is unwise, since the deeper dental lesions may be simulated by other local conditions, and an abscess which actually exists may not be shown. The local and clinical features are to be noted carefully, and extreme caution should be observed in ascribing a systemic infection to the mouth condition. Bacteriologic study is essential in oral infections. Sanitary oral cavities are potent factors in limiting the incidence and spread of communicable diseases.

### ROENTGEN-RAY STUDIES OF DENTAL DEFECTS

*Henry K. Pancoast*

The roentgenologist is not able to judge of the direct connection between dental conditions and their supposedly resultant systemic conditions. This is the work of the internist. Not all "clear areas" are due to abscesses at the roots of teeth. Some may be due to old infections, others to chemical irritants. If read properly, however, most of these "clear areas" represent active abscesses. *I am*

*opposed to the wholesale extraction of teeth.* In many instances it is possible to treat abscessed teeth successfully and have good service from these teeth afterward. An abscess may be present at the root of a tooth and not show in the roentgenogram, but that is a rare occurrence. Experience in the interpretation of the roentgenograms is more important than the technic, and such interpretation is best carried out by the dentist and the roentgenologist in collaboration.—*A. M. A. Journal,* Feb. 14, 1920.

